# Declining Medicare Work Relative Value Unit Reimbursement in Orthopaedic Surgery: A Cross-Specialty Analysis from 2000 to 2025, with Projections to 2030

**DOI:** 10.2106/JBJS.OA.26.00064

**Published:** 2026-07-13

**Authors:** Owen Falkenberg, Omar Badr, Davis Le, Cyrus Wong, Nicholas Smith, David Shau

**Affiliations:** 1 Burnett School of Medicine at TCU, Fort Worth, Texas; 2 Texas Hip and Knee Center, Fort Worth, Texas; 3 Texas Health Neurosurgery and Spine Specialists, Fort Worth, Texas; 4 Mount Sinai Medical Center, Miami Beach, Florida

## Abstract

**Background:**

Inflation-adjusted Medicare reimbursement has declined over the past 2 decades, raising concerns about the sustainability of procedural practice and patient access to care. Prior trend analyses are often limited to single specialties or use aggregate payments, which can obscure changes in the valuation of surgeon work. We sought to assess reimbursement trends of orthopaedic surgery and compare with other surgical specialties, using work Relative Value Units (wRVUs).

**Methods:**

Using the Centers for Medicare & Medicaid Services Physician Fee Schedule and the Medicare Physician & Other Practitioners by Provider and Service data set, we identified 10 of the most performed procedures in 2023 for orthopaedic surgery, neurosurgery, general surgery, and urology. Reimbursement was calculated from wRVUs using annual conversion factors, inflation-adjusted to January 2025 US dollars, and evaluated from 2000 to 2025 with projections to 2030.

**Results:**

Orthopaedic surgery demonstrated the greatest absolute reimbursement decline, decreasing an average of $526.47 per procedure (50.27%). The remaining declines were $454.14 (52.31%, neurosurgery), $298.94 (46.47%, general surgery), and $242.47 (52.01%, urology), with negative compound annual growth rates across all fields. Projections suggest continued decline through 2030, with General Surgery and orthopaedic surgery exhibiting the largest projected proportional reduction (65.46% and 64.67%, respectively) but orthopaedic surgery demonstrating the greatest projected absolute reimbursement decline by 2030, exceeding $150 per procedure.

**Conclusions:**

The valuation of surgeon work has eroded broadly across procedural medicine with orthopaedic surgery experiencing the worst reimbursement reductions, emphasizing the need for collaboration and advocacy among surgical proceduralists. These system-wide trends raise concerns about workforce sustainability, access to complex surgical care, and the effectiveness of specialty specific advocacy efforts to reimbursement policy.

**Level of Evidence:**

Level IV, economic. See Instructions for Authors for a complete description of levels of evidence.

## Introduction

Over the past 2 decades, inflation-adjusted Medicare reimbursement has declined, despite rising health expenditures, increasing practice overhead, and growing procedural and administrative burden^1-3^. Orthopaedic surgery is one of the largest procedural specialties in Medicare and can be sensitive to reimbursement pressures due to high procedural volume and intensity^3^. The cumulative effect reflects a broader devaluation of procedural work that has implications for workforce stability and patient access across surgical care, especially in orthopaedic surgery.

The Medicare Relative Value Unit (RVU) framework remains the mechanism by which the Centers for Medicare & Medicaid Services (CMS) quantifies physician services. RVUs composed of wRVUs, practice expense RVUs, and malpractice RVUs^4,5^. In principle, wRVUs represent the physician’s time, skill, training, and cognitive effort^5^. However, growing literature suggests that wRVUs may not fully capture operative complexity and time for modern procedures, particularly as technology adoption and case complexity increase^6-9^.

Many reimbursement trend studies report total RVUs or total payments, integrating components influenced by regional cost structures, site-of-service dynamics, and institutional overhead^9,10^. While informative, this combined approach can mask changes in how surgeon work is valued. A wRVU-isolated analysis provides a lens on the valuation of surgical expertise and effort, independent of facility and liability adjustments.

While specialty-specific analyses have documented declines within individual fields, fewer studies have compared wRVU-derived reimbursement trajectories across surgical specialties^11,12^. In the setting of conversion-factor reductions and budget-neutrality mechanics, fragmented specialty-by-specialty advocacy may be less effective than coordinated attention to the erosion of procedural work. Accordingly, the purpose of this study was to evaluate long-term trends in inflation-adjusted wRVU-based reimbursement for common orthopaedic procedures, compare with other surgical specialties from 2000 to 2025, and project through 2030.

These trends occur within a reimbursement framework constrained by budget neutrality, where upward revaluation of certain services is frequently offset by downward adjustments elsewhere. This creates a zero-sum environment in which specialties compete for a fixed pool of physician payment, and incremental reductions to high-volume, established procedures can accumulate over time.

## Materials and Methods

### Race and Ethnicity Omission Statement

This paper does not report any demographic information due to its focus on reimbursement trends, justifying the omission of race and ethnicity from outcomes.

Four surgical specialties were selected for comparison: orthopaedic surgery, neurosurgery, urology, and general surgery. Specialties in which common procedure codes are frequently performed in non-CMS reimbursement settings, such as ophthalmology, were excluded.

Using “The Medicare Physician & Other Practitioners by Provider and Service data set,” 10 of the most commonly performed procedures in the year 2023 were selected for each specialty by procedure count. Selecting the most commonly performed procedures allowed the analysis to reflect reimbursement trends affecting a significant proportion of Medicare beneficiaries. Each procedure’s Current Procedural Terminology (CPT) code was investigated on the CMS Physician Fee Schedule, and the annual wRVU data were obtained from 2000 to 2025. The wRVU was selected as the sole investigative variable because it isolates physician work valuation over time, while malpractice and facility fee RVUs were excluded. The procedures analyzed are listed in Appendix 1, with procedure-level data in Appendix 2 (Appendix 1, 2).

CPT substitutions were required for 3 procedures:

CPT 29827 used CPT 23412 from 2000 to 2002 before wRVU data became available in 2003.
CPT 19303 used CPT 19180 from 2000 to 2006 before wRVU data became available in 2007.
CPT 64561 was analyzed beginning in 2002 because no comparable code with earlier wRVU data was available.

The annual reimbursement for each CPT code was calculated by multiplying the wRVU units by the corresponding annual conversion factor (CF). The CFs were obtained from a CMS data sheet provided by the AMA^13^. In years with multiple CFs, a weighted annual average was calculated. The monetary value was then inflation-adjusted to January 2025 USD using the Bureau of Labor Statistics CPI Inflation Calculator^14^.

Reimbursement trends were summarized using percent change and compound annual growth rate (CAGR) from 2000 to 2025 and from 2000 to projected 2030 values^15^. Monetary values were plotted with lines of best fit, and linear and second-order polynomial models were generated for each procedure, with model selection based on R^2^. The second-order polynomial models provided the best fit across procedures and were used to illustrate potential reimbursement trajectories under the current reimbursement structure rather than to predict future reimbursement values.

## Results

**Fig. 1:**
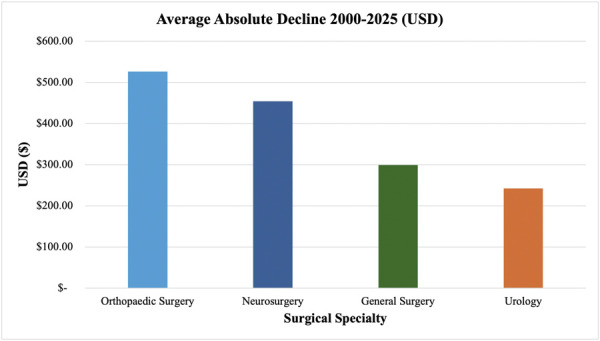
Absolute inflation-adjusted Medicare work RVU reimbursement declines by surgical specialty (2000-2025). RVU= relative value units.

All specialties analyzed from 2000 to 2025 displayed a negative CAGR and decline in absolute reimbursement with orthopaedic surgery experiencing the greatest, averaging a decline of $526.47 per procedure (Fig. 1). Similar findings were seen in the projection to 2030. Reimbursement values of each specialty were averaged per year, followed by the calculation of reimbursement declines and CAGRs from these averages. A supplementary analysis of CMS procedural volume from 2009 to 2024 demonstrated increased volume for analyzed procedures; however, incomplete data before 2009 precluded analysis across the full study period (Supplementary Table 1).

### Orthopaedic Surgery

Across the selected 10 most commonly performed orthopaedic surgeries in 2023, the average decline in reimbursement from 2000 to 2025 was from $1038.87 to $512.40, representing a 50.27% decline, producing a CAGR of −2.78%. Using projections, the average absolute reimbursement is predicted to decrease to $361.66, representing a 64.67% decline and producing an average CAGR of −3.45% from 2000 to 2030.

### Neurosurgery

Across the selected 10 most commonly performed neurosurgical surgeries in 2023, the average decline in reimbursement from 2000 to 2025 was from $875.84 to $421.70, representing a 52.31% decline, producing a CAGR of −2.95%. Using projections, the average absolute reimbursement is predicted to decrease to $360.70, representing a 59.46% decline and producing an average CAGR of −2.98% from 2000 to 2030.

### General Surgery

Across the selected 10 most commonly performed general surgeries in 2023, the average decline in reimbursement from 2000 to 2025 was from $669.48 to $370.54, representing a 46.47% decline, producing a CAGR of −2.59%. Using projections, the average absolute reimbursement is predicted to decrease to $220.74, representing a 65.46% decline and producing an average CAGR of −3.64% from 2000 to 2030.

### Urology

Across the selected 10 most commonly performed urologic surgeries in 2023, the average decline in reimbursement from 2000 to 2025 was from $465.43 to $222.96, representing a 52.01% decline, producing a CAGR of −2.93%. Using projections, the average absolute reimbursement is predicted to decrease to $142.66, representing a 64.87% decline and producing an average CAGR of −3.69% from 2000 to 2030.

**Fig. 2:**
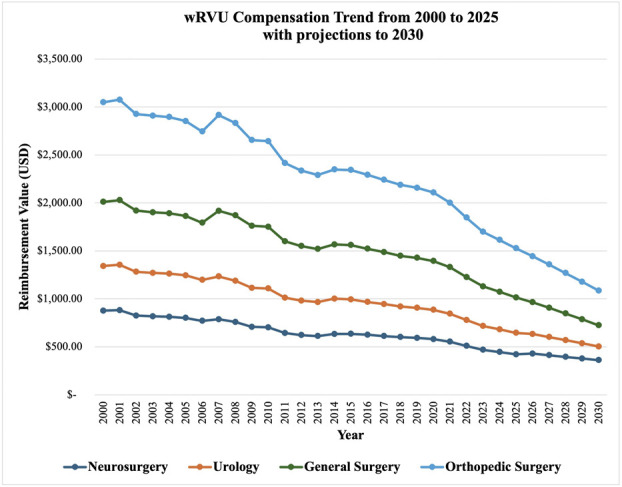
Inflation-adjusted work RVU-based Medicare reimbursement trends for 4 surgical specialties, averaged across the 10 most performed procedures per specialty, from 2000 to 2025 with projections to 2030 (2025 USD). RVU= relative value units.

Inflation-adjusted wRVU-derived reimbursement declined with negative growth rates observed across orthopaedic surgery, neurosurgery, general surgery, and urology (Table I and Fig. 2). These findings demonstrate consistent erosion across high-volume procedural work, with neurosurgery showing the greatest proportional decline (52.31%) and general surgery demonstrating the most severe projected trajectory (65.46%).

**TABLE I:** Inflation-Adjusted Medicare Work Relative Value Units Reimbursement Declines by Surgical Specialty (2000-2025)

	Neurosurgery	Urology	General Surgery	Orthopedic Surgery
Absolute decline to 2025 (USD)	$454.14	$242.47	$298.94	$526.47
Absolute decline to 2030 (USD)	$515.14	$322.77	$ 448.74	$677.21
% Decline to 2025	52.31%	52.01%	46.47%	50.27%
% Decline projected to 2030	59.46%	64.87%	65.46%	64.67%
CAGR to 2025	−2.95%	−2.93%	−2.59%	−2.78%
CAGR to 2030	−2.98%	−3.69%	−3.64%	−3.45%

CAGR = compound annual growth rates.

Values represent the mean percent decline among the 10 most commonly performed procedures per specialty from 2000 to 2025, with projected decline to 2030, corresponding compound annual growth rates, and absolute decline in USD.

## Discussion

This cross-specialty analysis demonstrates a sustained decline in inflation-adjusted Medicare reimbursement attributable to surgeon work (wRVUs) across orthopaedic surgery, neurosurgery, general surgery, and urology, with orthopaedic surgery experiencing the greatest absolute decline from 2000 to 2025 ($526.47), and the greatest projected absolute decline to 2030 ($677.21) (Figs. 1 and 2). While prior literature has documented reimbursement erosion within individual fields, these findings demonstrate consistent negative growth trajectories across procedural medicine^11,16-19^. These data indicate that reimbursement erosion is not specialty-specific, but rather a system-level issue affecting procedural care broadly^3,12,20,21^.

A central contribution of this study is its isolation of the wRVU component of Medicare reimbursement. By isolating wRVUs, this study avoids the confounding effects of regional expenses and malpractice costs inherent in total RVU analyses^22^. This provides a clearer view of how CMS values surgeon expertise, technical effort, and cognitive work over time^23^. The magnitude and consistency of decline observed across specialties suggest that the economic unit intended to value surgical work itself has been progressively eroded.

These findings must be interpreted through the Relative Value Scale Update Committee (RUC) framework and statutory budget-neutrality requirements, where physician payment increases for one service necessitate reductions elsewhere^24^. In this redistributive environment, surgical specialties compete for limited reimbursement, often resulting in incremental valuation cuts for high-volume procedures such as joint arthroplasty. Furthermore, innovation does not reliably expand the physician payment base; instead, established procedures may experience incremental downward pressure^25^.

This structural dynamic helps explain why “bread-and-butter” operations (high-volume procedures that form the backbone of many practices) continue to experience real-dollar cuts despite increasing patient complexity, technology adoption, and perioperative decision-making burden^26,27^. The present data elucidate what many surgeons observe in practice: repeated incremental reductions compound over time and absent meaningful change in how physician work is valued and updated, continued erosion should be expected. Contemporary complaints across surgical and procedural fields increasingly cite misalignment between effort, responsibility, and reimbursement as a contributor to burnout and professional dissatisfaction^16,28,29^. Persistent real-dollar erosion of work valuation may contribute to workforce strain, earlier retirement, reduced clinical effort, or shifting away from the most complex and time-intensive procedures.

Downstream implications extend beyond physician compensation. For orthopaedic surgeons, sustained declines may influence practice behavior in ways that affect access and quality. Financial pressure may contribute to consolidation, narrowing of payer mix, or reduced willingness to accept lower-reimbursing plans such as Medicare^25,30^. These reimbursement pressures threaten the financial viability of independent orthopaedic practice and may contribute to increasing motivation to pursue employed positions, ancillary income opportunities or other revenue streams. In addition, the most resource-intensive, lower-margin procedures may become increasingly unsustainable, potentially constraining access for older and medically complex patients who rely on Medicare and placing pressure on the timely delivery of care.

These findings also question the efficacy of fragmented, specialty-specific advocacy in addressing reimbursement erosion. When declines are observed broadly across procedural medicine, system-level solutions may be required. Coordinated engagement among specialty-specific professional societies and other physician groups may better address conversion-factor volatility, budget-neutrality constraints, and sustained real-dollar reductions in surgeon work valuation. To prevent continuous redistribution of RVUs between procedural and nonprocedural physician specialties, we suggest a unified approach that address support for inflationary updates to the CF, continuous reassessment of budget thresholds, and reassessment of the methods in which value is assessed on high volume procedures.

Ultimately, this analysis suggests that continued erosion of wRVU-derived reimbursement represents a structural threat to procedural medicine. Continued monitoring of wRVU valuation trends, alongside objective assessment of procedural complexity, workforce capacity, burnout, and patient access, will be critical to determining whether current reimbursement models remain aligned with the realities of modern surgical care.

This study is subject to limitations. First, this analysis is limited to 4 surgical specialties and relied on the 10 most commonly performed procedures per specialty in 2023, which may not capture trends in less common or highly specialized services. In addition, this analysis only reflects Medicare reimbursement, not accounting for other components of surgeon compensation such as call pay, salary structures, or productivity incentives. Increasing procedural volume may offset declining reimbursement, but the 2009 to 2024 supplementary analysis was limited by incomplete data and should be interpreted as contextual evidence of volume offset in that procedural volume changes may not adequately cover reimbursement declines (Supplementary Table 1). Furthermore, future reimbursement figures were generated using a second-order polynomial line of best fit based on historical data from 2000 to 2025. These forecasts are highly sensitive to past trends and assume the underlying mechanisms that drive wRVU changes will continue their current trajectory. Because this analysis is limited to Medicare reimbursement, the findings may not apply to private insurers, self-pay patients, or health systems that administer their own insurance. Future legislative interventions, RUC methodology changes, or unexpected economic shifts all potentially alter the decline significantly, although there are no signals of change to date. Finally, in years where the CMS enacted midyear legislative adjustments, a weighted average of the CF was necessarily used. While this standardizes the monetary calculation, it represents an approximation of the average reimbursement across the entire calendar year.

Future studies should expand upon these findings to explore more nuanced aspects of the reimbursement crisis. By comparing wRVU reimbursement trends against objective metrics such as surgeon’s reported operative time, incidence of comorbidities, and surgical approach modifications, we may be able to explore if the decline in wRVU value is being compounded by an increase in the effort required by the surgeon to perform the procedure. In addition, future studies should investigate how the decrease in wRVU reimbursement interacts with regional variations in Practice Expense and Malpractice RVUs. This would explore differences in how these economic trends are impacting surgeons in rural vs. urban settings, as well as independent vs. hospital-employed practices. For orthopaedic surgeons, these findings emphasize the importance of continued evaluation of physician work, as reimbursement erosion may influence workforce sustainability and patient access to orthopaedic care.

## Conclusion

This study extends prior specialty-specific analyses and highlights the systemic nature of physician work devaluation by demonstrating reimbursement erosion across multiple procedural specialties using a wRVU-based framework. While orthopaedic surgery experienced the worst absolute decline, declines were observed consistently among various surgical specialties and projected to continue. If sustained, these trends may exacerbate surgeon burnout, strain workforce capacity, and constrain patient access to care. Future work should assess the relationship between reimbursement decline and fellowship application trends to better understand how reimbursement erosion may influence orthopaedic subspeciality participation. These findings suggest continued reliance on the current valuation framework warrants reassessment if procedural capacity, workforce stability, and patient access are to be preserved.

## Funding

No external funding was received for this study.

## Ethical Approval

IRB: Exempt (publicly available CMS data sets).

## Appendix

Supporting material provided by the authors is posted with the online version of this article as a data supplement at jbjs.org (http://links.lww.com/JBJSOA/B264). This content was not copyedited or verified by JBJS.
